# Problem Gambling Among Adolescents in Uganda: A Cross-sectional Survey Study

**DOI:** 10.1007/s10899-023-10205-2

**Published:** 2023-04-08

**Authors:** Michael U. Anyanwu, Zsolt Demetrovics, Mark D. Griffiths, Zsolt Horváth, Andrea Czakó, Francis Bajunirwe, Imelda Tamwesigire

**Affiliations:** 1 Department of Community Health, Mbarara University of Science and Technology, P.O Box 1410, Mbarara, Uganda; 2 Centre of Excellence in Responsible Gaming, University of Gibraltar, Gibraltar, Gibraltar; 3 Institute of Psychology, ELTE Eötvös Loránd University, Budapest, Hungary; 4 International Gaming Research Unit Psychology Department, Nottingham Trent University, Nottingham, UK

**Keywords:** Gambling, Adolescent gambling, Problem gambling, Adolescence, Uganda

## Abstract

In recent years, gambling has evolved and grown substantially with new gambling activities and facilities being introduced, making gambling products and opportunities more available than ever before in Uganda. While the growth of gambling industry is considered to have a beneficial impact on the economy, it is increasingly becoming a social and public health issue especially among a minority of young people who experience problem gambling, which can damage personal, family, vocational, and academic pursuits. The present study estimated the prevalence of problem gambling and identified the socio-demographic, school, environmental and health risks of problem gambling among secondary school students in Mbarara Municipality, Uganda. A cross-sectional study was conducted among secondary school students in Mbarara Municipality. A total of 921 students from 12 schools were recruited using cluster sampling. An ordinal logistic regression model was used to explore the relationship between problem gambling and the socio-demographic, academic, environmental and health variables. Of 905 participants, 362 reported having ever gambled (40%), and 160 were classified as problem gamblers (17.7%; 44.2% among those who had ever gambled). Problem gambling was significantly associated with being male, being non-religious, other religion (African traditional religion), having employment (outside of school), distance to nearest gambling venue, parental gambling, peer gambling, substance use, risky sexual behavior, and psychological distress. The present study found a very high prevalence of problem gambling among Ugandan secondary school students. Therefore, there is need to institute public health measures towards raising awareness, prevention and treatment of problem gambling among Ugandan adolescents.

## Introduction

Problem gambling is a gambling behavior that compromises, disrupts or damages personal, family, academic and vocational pursuits (Derevensky, 2012; Hardoon et al., 2004; Neal et al., 2005). Any excessive gambling activity could result in negative consequences such as financial problems, and physical and mental health issues, as well as impairment of family and community relationships (Abbott et al., 2015). The term ‘problem gambling’ is sometimes used interchangeably with ‘compulsive gambling’, ‘irresponsible gambling’, ‘harmful gambling’, ‘pathological gambling’, ‘addictive gambling’ and ‘gambling disorder’ by those in the media (and occasionally by scholars in the gambling studies field). However, these terms are more accurately used to describe different levels of severity and frequency of gambling. Gambling disorder is one of the more extreme forms of gambling, and is defined by criteria outlined in the latest (fifth edition) of the *Diagnostic and Statistical Manual of Mental Disorders* (DSM-5) while terms such as ‘problem gambling’ may not fulfil the diagnostic criteria for gambling disorder (Abbott et al., 2015; American Psychiatric Association, 2013).

Adolescents are increasingly exposed to gambling due to growth of the gambling industry over the years as well as weak gambling regulations in some countries (Abdi et al., 2015; Derevensky et al., 2004). They are more likely to experience problem gambling compared to other cohorts due to their tendency to take risks, their poor coping skills, and being unaware of the negative consequences of gambling (Burnett et al., 2010; Gupta & Derevensky, 2000; Montiel et al., 2021; Steinberg, 2008). Studies have shown that the prevalence of problem gambling among adolescents is two to four times higher than that of adults (Dowling et al., 2017; Huang & Boyer, 2007; Huang et al., 2007; Jacobs, 2000). A meta-analysis of prevalence surveys reported 3% of problem gambling among adults (Stucki & Rihs-Middel, 2007) and systemic review of problem gambling indicated a range of 0.12–5.8% worldwide (Calado & Griffiths, 2016). However among adolescents, a meta-analysis of the prevalence rates of problem gambling showed that the mean rate of problem gambling was 10.2% with country-specific rates also being reported: US (3%), Canada (8.7%), Singapore (8.7%), Scotland (3.9%), Japan (4.2%), Nigeria (14.2%) and China (6.4%) (Nowak & Aloe, 2014).

Previous studies have identified several adverse outcomes associated with problem gambling among adolescents. These include increased delinquency and crime (Kryszajtys et al., 2018 Räsänen et al., 2015; Richard, 2013), disrupted family and peer relationships (Derevensky, 1999; Uwiduhaye et al., 2021), poor academic performance (George et al., 2016), financial problems (Livazović & Bojčić, 2019), substance use (Jaisoorya et al., 2017; Nabifo et al., 2021), risky sexual behavior (Kiragga et al., 2021; Martins et al., 2014), suicidal ideation and attempts (Kaggwa et al., 2022; Petry & Kiluk, 2002; Wardle & McManus, 2021), and physical and mental health issues (Fong, 2005; Hardoon et al., 2004).

Researchers have shown that several factors predispose adolescents to problem gambling among which are socio-demographic and environmental influences. Demographic characteristics such as young age, male gender, being employed, and not being religious have been associated with problem gambling in this population (Abdi et al., 2015; George et al., 2016; Hoffman, 2000; Matama, Mbago, & Ngoboka, 2021; Nowak, 2018). Furthermore, extant studies have reported specific socio-cultural factors including peer gambling, parental gambling, and societal perception on gambling as being associated with problem gambling (Freund et al., 2019; Keatley et al., 2019; Matthew et al., 2021; Riley et al., 2021). Among other environmental factors, gambling advertisements and accessibility have been closely associated with problem gambling among adolescents (Bozzato et al., 2020; Monaghan et al., 2008; Riley et al., 2021; Wonju et al., 2020).

Sub-Saharan Africa has one of the world’s youthful populations (Ashford, 2007). However, few studies have been conducted on problem gambling among adolescents in this region (Bitanihirwe & Ssewanyana, 2021). For example, despite Uganda having a very young population with more than 60% of the population being aged 24 years and below (United Nations Population Fund, 2017), few studies on problem gambling among this population exists. In Uganda, the gambling industry has experienced a rapid increase since 2000 with different gambling types and facilities. The proliferation of gambling has seen the industry diversify from the early gambling activities such as casino gambling to new gambling activities like sports betting and online gambling. In 2014 in Uganda, there were estimated to be over 1000 gambling outlets mostly for sports betting as well as other outlets for national lottery/lotto games, casino gambling, slot machines and pool betting (Ahaibwe et al., 2016; Masaba & Blaszynski, 2016; Ssewanyana & Bitanihirwe, 2018). As a result of the expansion of the gambling industry and advancement in technology, most of the aspects of the gambling law outlined in the Ugandan National Lotteries Act of 1967 became outdated (Ministry of Finance Planning and Economic Development [MFPED], 2013). Therefore, the National Lotteries Board (NLB) was rebranded and redefined as the National Gaming Board (NGB), operating under the Lotteries and Gaming Act of 2016. According to the Ugandan Act, a minor is a person who is aged below 25 years, and the Act incriminates operators from accepting payments from a minor or granting them access to gambling premises (Government of Uganda, 2016). However, gambling laws are rarely implemented in Uganda due to weak regulatory framework and lack of capacity of the NGB to regulate all gambling activities in the country (Herskowitz, 2021; Matama et al., 2021; MFPED, 2013).

Despite the growth of the gambling industry and weak regulations in Uganda, there are few studies on adolescent problem gambling in the country. Therefore, the present study aimed to estimate the prevalence of problem gambling, identify socio-demographic, academic and environmental factors associated with problem gambling, as well as assess the behavioral, psychological and chronic health risks associated with problem gambling in school-aged adolescents in Uganda.

## Methods

### Study Participants and Procedure

A cross-sectional study was conducted among secondary school students in Mbarara Municipality, Uganda. Mbarara Municipality is a town located in South Western part of Uganda. It is the main municipal, administrative and commercial center of Mbarara District. According to the list of schools obtained from Mbarara Municipal Education Office, there were 35 secondary schools in Mbarara Municipality with a total enrollment of 11,106 students (Ministry of Education and Sports, 2016; Uganda Bureau of Statistics, 2017).

Multi-stage cluster sampling was used in the selection of secondary school students. In order to have a representative sample of the secondary school students in Mbarara Municipality, secondary schools were randomly selected from all the six Divisions in the Municipality. Secondary schools located at each Division were listed and categorized into public or private schools. Two schools (one private and one public) were randomly selected from each Division. The final stage involved the random selection of students in each of the secondary schools. At each school, at least 60 students were randomly selected with ten students from each class (schools in Uganda have a total of six classes) using the class register.

The secondary schools sampled comprised both ordinary levels (four years of lower secondary school) and advanced levels (two years of upper secondary school). A total of 921 students from 12 schools were selected using a proportion of 10.2%, 95% confidence interval, 3% margin of error and design effect of 1.826. The study recruited students that had spent at least two months or one academic term at the school. The formula for random cluster sampling was used to determine the sample size. The sample size was calculated by Kish Leslie formulae and then multiplied by the design effect (Christie et al., 2009).

A self-administered survey was used to collect the data. Trained research assistants explained the objectives of the study to the students and assured them that all their information was anonymous, that there was no right or wrong answers, and that answers would not affect their grades in any way. They gave the survey to students that consented to the study and no incentive was given for participating in the study.

### Measures

A self-administered, structured survey was used to collect data from the students. Socio-demographic characteristics of the participants were collected: age, gender, religion, employment status, and type of school attended. Questions also assessed if they have been taught about gambling and its effect in school (yes/no), ever failed a subject in school exams (yes/no), exposure to gambling adverts (never/rare/sometimes/always), nearest gambling or betting centers (less than 1 km, 1-3 km, 3-5 km and above 5 km), and peer and parent involvement in gambling (yes/no).

#### Gambling and Problem Gambling

The gambling behavior of the students was assessed by the question “Have you ever gambled?” (yes/no). The Diagnostic Statistical Manual IV (Multiple Response format) adapted for Juveniles (DSM-IV-MR-J) was used to screen students that had ever gambled for possible problem gambling (Fisher, 2000). The tool assesses symptoms associated with problem gambling, including preoccupation, tolerance, loss of control, withdrawal symptoms, escaping problems, chasing losses, lies, and illegal acts, as well as family and academic disruptions. The tool has demonstrated adequate levels of validity and reliability and has been recommended for prevalence studies examining adolescent gambling. Scores on the DSM-IV-MR-J were used to classify participants as non-problem gamblers or problem gamblers. Participants with a score of 0–3 were classified as “non-problem gamblers” and those with a score of four and above were classified as “problem gamblers” (Abdi et al., 2015; Fisher, 2000; Odame et al., 2021). The Cronbach’s alpha in the present study was 0.75.

#### Substance Use

Substance use among the students was assessed using the Alcohol, Smoking and Substance Involvement Screening Test (ASSIST), a tool validated by World Health Organization for assessing the use of alcohol, tobacco products and other drugs (WHO ASSIST Working Group, 2002). ASSIST has demonstrated adequate levels of reliability and validity and is recommended for use in developing countries (Hides et al., 2009; Humeniuk & Ali, 2006). The present study used the second question *“In the past three months, how often have you used the substances mentioned (tobacco products, alcoholic beverages, cannabis, amphetamine type stimulants, inhalants, sedatives, hallucinogens, opioids and other substances?”* to assess substance use. A participant that had never used any of the substances was classified as having “no substance use” and those that had used any of the substances were classified as having “substance use”.

#### Psychological Distress

Psychological distress was evaluated with Kessler Psychological Distress Scale (K10) (Kessler et al., 2002), a ten-item scale with items rated on a five-point Likert scale that evaluates the frequency of depression and anxiety symptoms over the past four weeks. Psychological distress was categorized into normal, mild, moderate or severe distress. Scores range from 10 to 50, with score of 0–19 categorized as normal, 20–24 as mild distress, 25–29 as moderate distress and scores above 29 categorized as severe psychological distress. The K10 has been shown to have good psychometric properties in studies conducted in Africa (Andersen et al., 2011; Baggaley et al., 2007; Vissoci et al., 2018). The Cronbach’s alpha in the present study was 0.86.

#### Risky Sexual Behavior

The sexual behavior of the students was assessed with questions concerning being sexually active, condom use, and ever being pregnant. Risky sexual behavior was categorized as either “no risky sexual behavior” or “risky sexual behavior”. Any student that answered “no” to being sexually active or “yes” to being sexually active but “no” to her or his sexual partner ever being pregnant and “yes” to condom use were categorized as “no risky behavior”.

#### Chronic Illness

The chronic illness condition of the participants was determined by the question *“Do you have a chronic (long lasting) illness or health condition?”* with “yes” or “no” response.

### Statistical Analysis

Data collected from the surveys were inspected for errors. After inspection and cleaning, the questions were coded, entered, and analyzed using STATA 14. Incomplete surveys on gambling were excluded from the analysis. The characteristics of the participants were summarized using frequencies and percentages. The prevalence of problem gambling was obtained from the percentage of students that scored 4 or more on the DSM-IV-MR-J. Logistic regression was used to explore the bivariate association between problem gambling and the socio-demographic, academic, environmental, and health variables. All variables with a *p* < 0.05 in the univariate analysis were included in the multivariable ordinal logistic regression model. All tests were two-tailed and *p* < 0.05 was used as the level of significance.

## Results

### Sample Description

A total of 16 participants (1.7%) were excluded from the analysis due to incomplete information on gambling. The participants in the present study were aged 11–25 years (mean age: 16.9 years, SD = 2.2). The majority of the participants were aged below 18 years, (59.2%), male (53.8%), Catholics (37.1%), and attended private schools (54.4%). Table 1 presents the baseline characteristics of the participants.

**Table 1:** Baseline characteristics of the students

Variable	Non-gamblers n (%)	Non-problem gamblers n (%)	Problem gamblers n (%)	Total N = 905 (%)
Age				
Below 18 years	303 (61.8)	119 (64.0)	59 (43.1)	481 (59.2)
18 years and above	187 (38.2)	67 (36.0)	78 (56.9)	332 (40.8)
Gender				
Female	286 (53.0)	88 (43.8)	41 (26.0)	415 (46.2)
Male	254 (47.0)	113 (56.2)	117 (74.0)	484 (53.8)
Class in school				
S1	82 (15.2)	27 (13.4)	16 (10.0)	125 (13.9)
S2	69 (12.8)	39 (19.3)	21 (13.1)	129 (14.3)
S3	110 (20.4)	44 (21.8)	26 (16.3)	180 (20.0)
S4	132 (24.4)	36 (17.8)	30 (18.8)	198 (22.0)
S5	81 (15.0)	29 (14.4)	37 (23.1)	147 (16.3)
S6	66 (12.2)	27 (13.4)	30 (18.8)	123 (13.6)
Type of student				
Boarding	400 (75.3)	149 (74.1)	124 (80.0)	673 (75.9)
Day	131 (24.7)	52 (25.9)	31 (20.0)	214 (24.1)
Type of school				
Private	316 (58.2)	95 (47.0)	81 (50.6)	492 (54.4)
Public	227 (41.8)	107 (53.0)	79 (49.4)	413 (45.6)
Mixed-sex	477 (87.9)	159 (78.7)	148 (92.5)	784 (86.6)
Single-sex	66 (12.1)	43 (21.3)	12 (7.5)	121 (13.4)
Religion				
Catholic	188 (34.9)	75 (37.5)	70 (44.0)	333 (37.1)
Islam	54 (10.0)	17 (8.5)	12 (7.6)	83 (9.2)
Protestant	111 (20.6)	42 (21.0)	20 (12.6)	173 (19.3)
Pentecostal	134 (24.9)	33 (16.5)	29 (18.2)	196 (21.8)
Others	42 (9.7)	33 (16.5)	28 (17.6)	113 (12.6)
Part time job/Paid work				
No	444 (89.3)	164 (86.3)	126 (80.8)	734 (87.1)
Yes	43 (10.7)	26 (13.7)	30 (19.2)	109 (12.9)

### Prevalence of Problem Gambling

From the 905 participants, 543 reported that they had never gambled (60%), and 362 reported that they had ever gambled (40%). Based on the DSM-IV-MR-J scores, 160 were classed as problem gamblers (17.7%) while 202 were classed as non-problem gamblers (22.3%). Among the problem gamblers, seven students scored ‘8’ (0.8%), eleven scored ‘7’ (1.2%), 29 scored ‘6’ (3.2%), 58 scored ‘5’ (6.4%) and 55 scored ‘4’ (6.1%).

### Factors Associated with Problem Gambling

The univariate logistic regression showed that age, gender, religion, type of school employment, distance to nearest betting center, gambling advertisements, peer gambling, parental gambling, risky sexual behavior, substance use, and psychological distress were significantly associated with problem gambling (Table 2).

**Table 2:** Factors associated with problem gambling

Variable	UOR (95% CI)	*p-*value	AOR (95% CI)	*p-*value
Age				
Below 18 years	Ref.	< 0.001	Ref.	0.136
18 years and above	1.48 (1.12–1.96)		1.32 (0.92–1.90)	
Gender				
Female	Ref.	< 0.001	Ref.	0.001***
Male	2.16 (1.65–2.82)		1.80 (1.27–2.55)	
Type of student				
Day	Ref.	0.394	-	-
Boarding	1.13 (0.83–1.53)			
Type of school				
Private	Ref.	0.014	Ref.	0.502
Public	1.40 (1.08–1.82)		1.13 (0.79–1.63)	
Mixed-sex	Ref.	< 0.001	Ref.	0.654
Single-sex	1.06 (0.74–1.52)		1.13 (0.65–1.97)	
Religion				
Catholic	1.66 (1.15–2.38)		1.25 (0.80–1.96)	0.321
Islam	1.12 (0.66–1.92)	0.003	1.10 (0.56–2.19)	0.781
Protestant	1.12 (0.73–1.70)		1.02 (0.61–1.70)	0.937
Pentecostal	Ref.		Ref.	
Others	2.36 (1.50–3.71)		2.12 (1.18–3.81)	0.012*
Part time job/Paid work				
No	Ref.	0.020	Ref.	0.043*
Yes	1.69 (1.15–2.47)		1.64 (1.02–2.64)	
Distance to betting center				
Less than 1 km	2.53 (1.77–3.61)		1.69 (1.09–2.62)	0.018*
1–3 km	2.06 (1.36–3.11)	< 0.001	1.48 (0.89–2.45)	0.127
3–5 km	1.63 (0.98–2.70)		1.29 (0.69–2.42)	0.426
Above 5 km	Ref.		Ref.	
Gambling advert exposure				
Always/sometimes	1.87 (1.32–2.66)	0.001	1.00 (0.63–1.59)	0.994
Rare/never	Ref.		Ref.	
Gambling education in school				
No	Ref.			
Yes	1.30 (0.91–1.85)	0.297	-	-
Exam failure				
No	Ref.	0.194	-	-
Yes	1.18 (0.85–1.63)			
Peer gambling				
No	Ref.	< 0.001	Ref.	< 0.001***
Yes	5.32 (3.70–7.73)		3.26 (2.03–5.24)	
Parental gambling				
No	Ref.	< 0.001	Ref.	< 0.001***
Yes	2.48 (1.79–3.44)		2.14 (1.44–3.20)	
Chronic illness				
No	Ref.	0.818	-	-
Yes	1.07 (0.80–1.44)			
Substance use				
No	Ref.	< 0.001	Ref.	0.004**
Yes	2.87 (2.19–3.76)		1.64 (1.17–2.31)	
Risky sexual behavior				
No	Ref.	< 0.001	Ref.	0.001**
Yes	2.33 (1.69–3.22)		1.95 (1.31–2.91)	
Psychological distress				
Normal	Ref.		Ref.	
Mild	1.58 (1.14–2.21)	< 0.001	1.02 (0.68–1.55)	0.920
Moderate	2.39 (1.66–3.43)		1.35 (0.86–2.13)	0.195
Severe	1.96 (1.32–2.92)		1.83 (1.11–3.02)	0.018*

UOR: Unadjusted odds ratio (represents the uniivariate associations between the variables) AOR: Adjusted odds ratio (represents the regression coefficients from the multivariable model)

ref: Reference category;

*p < 0.05; **p < 0.01; ***p < 0.001

In the multivariable ordinal logistic regression analysis, significant and positive predictors of problem gambling included being male (AOR [95% CI}=1.80 [1.27; 2.55]), being non-religious/other religion (AOR [95% CI] = 2.12 [1.18; 3.81]), engaging in paid work or part time job (AOR [95% CI] = 1.64 [1.02; 2.64]), having a distance less than one kilometer to the nearest gambling center (AOR [95% CI] = 1.69 [1.09; 2.62]), peer gambling (AOR [95% CI] = 2.14 [1.44; 3.20]) and parental gambling (AOR [95% CI] = 2.14 [1.44; 3.20]). The odds of being a problem gambler were 1.64 higher among students that used psychoactive substances (95% CI = 1.17; 2.31). Students that had engaged in risky sexual behavior were 1.95 times more likely to be problem gamblers compared to those who did not (95% CI = 1.31; 2.91). Participants with severe psychological distress were more likely to be problem gamblers compared to those who reported normal psychological distress (AOR [95% CI] = 1.83 [1.11; 3.02]). Details of the bivariate and multivariate analysis are presented in Table 2.

## Discussion

The present study found the prevalence of problem gambling to be 17.7%. Previous studies have estimated different prevalence rates from various countries. In Uganda, while there is no study conducted on problem gambling among secondary school students, a study conducted among the adult population reported a prevalence rate of 5.7% (Ahaibwe et al., 2016). Nonetheless, it has been shown that prevalence of problem gambling is 2–4 times higher among adolescents compared to adults (Blinn-Pike et al., 2010; Valentine, 2008). Similar studies in Ethiopia reported that 7% of high school students in Addis Ababa were problem gamblers (Abdi et al., 2015). Lower rates of problem gambling among adolescents have been reported in the US (Huang et al., 2007; Lee et al., 2014), Australia (Freund et al., 2019), India (George et al., 2016; Jaisoorya et al., 2017), and the UK (Forrest & McHale, 2012) but a higher rate has been reported in a recent study in Ghana (Odame et al., 2021). The possible reasons that could account for these differences are the varying research methodologies and psychometric scale used in estimating problem gambling, distinct gambling laws and guidelines, socio-cultural and economic factors, and different study populations.

Among the demographic factors considered in the present study, gender was significantly associated with problem gambling with male students being more likely to report problem gambling compared to female students. This is consistent with previous studies which showed that males were at a greater risk of problem gambling than females (Abdi et al., 2015; Forrest & McHale, 2012; George et al., 2016; Jaisoorya et al., 2017; Merkouris et al., 2016). Students who were not religious or belong to other religions such as the African traditional religion had higher odds of being a problem gambler. Different religions have varying views on gambling, and polytheistic religions may have a positive view but monotheistic religions tend to disapprove gambling. This finding is in agreement with prior studies (Odame et al., 2021; Welte et al., 2008) although some studies report contrary results (Aguocha et al., 2019; Hoffman, 2000). There is overwhelming evidence that employed adolescents are more likely to gamble and more likely to be a problem gambler than those who are not employed (Ahaibwe et al., 2016; Forrest & McHale, 2012; George et al., 2016; Hing, Russell, Vitartas & Lamont, 2016). This is in line with the results of the present study where students that had part-time jobs or engaged in some paid work had higher odds of problem gambling. Earnings from work provides more money for students to spend on gambling (Gainsbury, 2012).

As in previous studies, the findings of the present study showed that the odds of problem gambling were higher among students who lived near gambling centers than those who lived further from gambling centers. The findings from existing studies are consistent with the present study where increased accessibility and availability of gambling products were associated with problem gambling (Abdi et al., 2015; Bozzato et al., 2020; Lawn et al., 2020; Riley et al., 2021).

Similar to previous studies (Abdi et al., 2015; Forrest & McHale, 2012; Freund et al., 2019; Riley et al., 2021), peer and parental gambling were associated with problem gambling among adolescents. Students whose parents and peers gambled had higher odds of problem gambling than those whose friends and parents did not engage in any gambling activity. Also, one study demonstrated that adolescents who believed that their parents gambled were more likely to try gambling themselves and had higher rates of problem gambling (Magoon & Ingersoll, 2006). Adolescents who experienced family members and peers participating in gambling may identify gambling as socially acceptable and less harmful (Campbell et al., 2011).

However, unlike the results of previous studies (George et al., 2016; Jaisoorya et al., 2017; Livazović & Bojčić, 2019; Odame et al., 2021), school factors (exam failure, gambling education and type of school) considered in the present study were not significantly associated with problem gambling. The difference in study findings could be due to population characteristics and methodology used in the study.

The present study confirmed most of the results from previous studies concerning health correlates of gambling. Substance use was found to be associated with problem gambling. The results are comparable to existing studies conducted among adolescents (Cheung, 2014; Freund et al., 2019; Huang et al., 2007). The association between substance use and problem gambling could be possible due to financial and social reasons. Adolescent problem gamblers tend to have some form of employment and/or engage in income-generating activities and can afford to buy psychoactive substances as well as have money for gambling. Also, they are more likely to drink alcohol, smoke cigarettes and use other psychoactive substances when they socialize with friends compared to non-gamblers who may isolate or engage in other activities when they are socializing with their peers. Furthermore, adolescents who were problem gamblers were more likely to engage in risky sexual behavior which is similar to previous studies (Kiwujja & Mugisha, 2019; Martins et al., 2014). Gambling is a high-risk behavior and adolescents that gamble have the tendency to engage in other risky behaviors. Also, participation in gambling activities exposes adolescents to environments where high risk behaviors are socially condoned or perceived as low risks. The presence of severe psychological distress among adolescents with problem gambling supports previous findings that there is an association between problem gambling and psychological distress (Abdi et al., 2015; Jaisoorya et al., 2017; Martin et al., 2014). Severe psychological distress among adolescent problem gamblers could be attributed to financial strain associated with gambling, adverse social circumstances, and reduced ability to cope with academic and routine daily activities. The association between problem gambling and chronic health conditions was not significant, although a previous study reported contrary results (Tracie et al., 2010).

### Strengths and Limitations

There are a number of limitations to the present study. The findings may not be generalized to the larger population of adolescents because the study was limited to school-going adolescents. Also, the study was cross-sectional which makes it difficult to draw causal relationship among the variables examined. In addition, the information was self-reported and the possibility of over-reporting by the students cannot be excluded. Although the DSM-IV-MR-J has been validated and used in low and middle-income countries, the scale may have over-estimated the prevalence of problem gambling in the present sample. However, on the other hand, to the best of present authors’ knowledge, this is the first study that has estimated the prevalence of problem gambling and identified the risk factors associated with problem gambling among school-aged adolescents in Uganda. The study used international instruments which are validated and have been used in developing countries for screening of problem gambling and other variables. Also, the study included a reasonably large number of participants selected by cluster sampling and drawn from different types of school and Divisions of Mbarara Municipality.

## Conclusions

The estimated prevalence of problem gambling in the present study was high and indicated that almost two out of every ten school-aged adolescents was at risk of problem gambling. The prevalence of problem gambling among secondary school students in Uganda is high relative to rates from other countries. The evidence from the present study underscores the need to institute and implement regulatory measures that will prevent problem gambling in order to improve the health and welfare of adolescents in Uganda.
